# Risk for cancer development in familial Mediterranean fever and associated predisposing factors: an ambidirectional cohort study from the international AIDA Network registries

**DOI:** 10.3389/fimmu.2024.1397890

**Published:** 2024-05-10

**Authors:** Antonio Vitale, Valeria Caggiano, Abdurrahman Tufan, Gaafar Ragab, Ezgi Deniz Batu, Piero Portincasa, Emma Aragona, Jurgen Sota, Giovanni Conti, Amato De Paulis, Donato Rigante, Alma Nunzia Olivieri, Ali Şahin, Francesco La Torre, Giuseppe Lopalco, Marco Cattalini, Maria Cristina Maggio, Antonella Insalaco, Petros P. Sfikakis, Elena Verrecchia, Derya Yildirim, Hamit Kucuk, Riza Can Kardas, Ahmed Hatem Laymouna, Mahmoud Ghanema, Moustafa Ali Saad, Seher Sener, Hulya Ercan Emreol, Seza Ozen, Nour Jaber, Mohamad Khalil, Agostino Di Ciaula, Carla Gaggiano, Giuseppe Malizia, Andrea Affronti, Serena Patroniti, Meri Romeo, Jessica Sbalchiero, Francesca Della Casa, Ilaria Mormile, Sara Silvaroli, Maria Francesca Gicchino, Neşe Çabuk Çelik, Maria Tarsia, Anastasios Karamanakos, José Hernández-Rodríguez, Paola Parronchi, Daniela Opris-Belinski, Patrizia Barone, Andreas Recke, Stefania Costi, Paolo Sfriso, Henrique A. Mayrink Giardini, Stefano Gentileschi, Ewa Wiesik-Szewczyk, Ibrahim Vasi, Roberta Loconte, Karina Jahnz-Różyk, Eduardo Martín-Nares, Jiram Torres-Ruiz, Alberto Cauli, Alessandro Conforti, Giacomo Emmi, Francesca Li Gobbi, Giovanni Rosario Biasi, Riccardo Terribili, Piero Ruscitti, Emanuela Del Giudice, Samar Tharwat, Antonio Luca Brucato, Benson Ogunjimi, Andrea Hinojosa-Azaola, Alberto Balistreri, Claudia Fabiani, Bruno Frediani, Luca Cantarini

**Affiliations:** ^1^ Department of Medical Sciences, Surgery and Neurosciences, Research Center of Systemic Autoinflammatory Diseases and Behçet’s Disease Clinic, University of Siena, Siena, Italy; ^2^ Azienda Ospedaliero-Universitaria Senese [European Reference Network (ERN) for Rare Immunodeficiency, Autoinflammatory and Autoimmune Diseases (RITA) Center], Siena, Italy; ^3^ Gazi University Hospital, Department of Internal Medicine, Division of Rheumatology, Ankara, Türkiye; ^4^ Internal Medicine Department, Rheumatology and Clinical Immunology Unit, Faculty of Medicine, Cairo University, Giza, Egypt; ^5^ Faculty of Medicine, Newgiza University, 6th of October City, Giza, Egypt; ^6^ Division of Pediatric Rheumatology, Department of Pediatrics, Hacettepe University School of Medicine, Ankara, Türkiye; ^7^ Clinica Medica “A. Murri”, Division of Internal Medicine, Department of Precision and Regenerative Medicine and Ionian Area (DiMePre-J), University of Bari Aldo Moro, Bari, Italy; ^8^ Division of Gastroenterology, Ospedali Riuniti Villa Sofia-Vincenzo Cervello, Palermo, Italy; ^9^ Pediatric Nephrology and Rheumatology Unit, Azienda Ospedaliera Universitaria (AOU), “G. Martino”, Messina, Italy; ^10^ Department of Translational Medical Sciences, Section of Clinical Immunology, University of Naples Federico II, Naples, Italy; ^11^ Center for Basic and Clinical Immunology Research (CISI), WAO Center of Excellence, University of Naples Federico II, Naples, Italy; ^12^ Department of Life Sciences and Public Health, Fondazione Policlinico Universitario A. Gemelli IRCCS, Rome, Italy; ^13^ Rare Diseases and Periodic Fevers Research Center, Università Cattolica del Sacro Cuore, Rome, Italy; ^14^ Department of Woman, Child and of General and Specialized Surgery, University of Campania “Luigi Vanvitelli”, Naples, Italy; ^15^ Division of Rheumatology, Department of Internal Medicine, Sivas Cumhuriyet University Medical Faculty, Sivas, Türkiye; ^16^ Department of Pediatrics, Pediatric Rheumatology Center, Giovanni XXIII Pediatric Hospital, University of Bari, Bari, Italy; ^17^ Department of Precision and Regenerative Medicine and Ionian Area (DiMePRe-J) Policlinic Hospital, University of Bari, Bari, Italy; ^18^ Pediatrics Clinic, University of Brescia and Spedali Civili of Brescia, [European Reference Network (ERN) for Rare Immunodeficiency, Autoinflammatory and Autoimmune Diseases (RITA) Center], Brescia, Italy; ^19^ University Department of Health Promotion, Mother and Child Care, Internal Medicine and Medical Specialties (PROMISE) “G. D’Alessandro”, University of Palermo, Palermo, Italy; ^20^ Division of Rheumatology, Ospedale Pediatrico Bambino Gesù, IRCCS [European Reference Network (ERN) for Rare Immunodeficiency, Autoinflammatory and Autoimmune Diseases (RITA) Center], Rome, Italy; ^21^ Joint Academic Rheumatology Program, Medical School, National and Kapodistrian University of Athens, [European Reference Network (ERN) for Rare Immunodeficiency, Autoinflammatory and Autoimmune Diseases (RITA) Center], Athens, Greece; ^22^ Department of Aging, Neurological, Orthopedic and Head and Neck Sciences, Fondazione Policlinico Universitario Agostino Gemelli Istituto di Ricovero e Cura a Carattere Scientifico (IRCCS), Rome, Italy; ^23^ Department of Pediatric Surgery, Fondazione Policlinico Universitario A. Gemelli IRCCS, Rome, Italy; ^24^ Clinical Pediatrics, Department of Molecular Medicine and Development, University of Siena, Siena, Italy; ^25^ Department of Rheumatology, “Evangelismos” General Hospital, Athens, Greece; ^26^ Department of Autoimmune Diseases, Institut d’Investigacions Biomèdiques August Pi I Sunyer (IDIBAPS), Hospital Clínic of Barcelona [European Reference Network (ERN) for Rare Immunodeficiency, Autoinflammatory and Autoimmune Diseases (RITA) Center], University of Barcelona, Barcelona, Spain; ^27^ Department of Experimental and Clinical Medicine, University of Florence, Florence, Italy; ^28^ Rheumatology and Internal Medicine Department, Carol Davila University of Medicine and Pharmacy, Bucharest, Romania; ^29^ Pediatric Rheumatology Unit, Department of integrated Maternal-Child and Reproduction Activity AOU “Policlinico-San Marco”, Catania, Italy; ^30^ Department of Dermatology, Allergology and Venerology, University Hospital Schleswig-Holstein, Lübeck, Germany; ^31^ Autoinflammatory and Autoimmune Diseases (RITA) Center, European Reference Network (ERN) for Rare Immunodeficiency, Lübeck, Germany; ^32^ Department of Clinical Sciences and Community Health, Research Center for Adult and Pediatric Rheumatic Diseases, University of Milan, Milan, Italy; ^33^ Rheumatology Unit, Department of Medicine, University of Padua, Padua, Italy; ^34^ Rheumatology Division, Faculdade de Medicina, Hospital das Clínicas, Universidade de São Paulo, São Paulo, Brazil; ^35^ Department of Internal Medicine, Pneumonology, Allergology and Clinical Immunology, Central Clinical Hospital of the Ministry of National Defense, Military Institute of Medicine, National Research Institute, Warsaw, Poland; ^36^ Department of Immunology and Rheumatology, Instituto Nacional de Ciencias Médicas y Nutrición Salvador Zubirán, Mexico City, Mexico; ^37^ Rheumatology Unit, Department of Medical Sciences, University and AOU of Cagliari, Cagliari, Italy; ^38^ Ospedale San Paolo di Civitavecchia, U.O. Medicina Generale, ASL Roma 4, Civitavecchia, Rome, Italy; ^39^ Department of Medical, Surgical and Health Sciences, University of Trieste, Italy, and Clinical Medicine and Rheumatology Unit, Cattinara University Hospital, Trieste, Italy; ^40^ Centre for Inflammatory Diseases, Department of Medicine, Monash Medical Centre, Monash University, Clayton, VIC, Australia; ^41^ Rheumatology Unit, Hospital S. Giovanni di Dio, Azienda USL-Toscana Centro, Florence, Italy; ^42^ Rheumatology Unit, Department of Biotechnological and Applied Clinical Sciences, University of L’Aquila, L’Aquila, Italy; ^43^ Pediatric and Neonatology Unit, Department of Maternal Infantile and Urological Sciences, Sapienza University of Rome, Latina, Italy; ^44^ Rheumatology and Immunology Unit, Internal Medicine Department, Mansoura University, Mansoura, Egypt; ^45^ Department of Internal Medicine, Faculty of Medicine, Horus University, New Damietta, Egypt; ^46^ Department of Biomedical and Clinical Sciences, Fatebenefratelli Hospital, Università di Milano, Milan, Italy; ^47^ Antwerp Unit for Data Analysis and Computation in Immunology and Sequencing, University of Antwerp, Antwerp, Belgium; ^48^ Antwerp Center for Translational Immunology and Virology, Vaccine and Infectious Disease Institute, University of Antwerp, Antwerp, Belgium; ^49^ Department of Pediatrics, Antwerp University Hospital, Antwerp, Belgium; ^50^ Center for Health Economics Research and Modeling Infectious Diseases, Vaccine and Infectious Disease Institute, University of Antwerp, Antwerp, Belgium; ^51^ Bioengineering and Biomedical Data Science Lab, Department of Medical Biotechnologies, University of Siena, Siena, Italy; ^52^ Ophthalmology Unit, Department of Medicine, Surgery and Neurosciences, University of Siena, Siena, Italy

**Keywords:** autoinflammatory diseases, FMF, tumor, neoplasm, rare diseases, treatment

## Abstract

**Objective:**

Inflammation has been associated with an increased risk for cancer development, while innate immune system activation could counteract the risk for malignancies. Familial Mediterranean fever (FMF) is a severe systemic inflammatory condition and also represents the archetype of innate immunity deregulation. Therefore, the aim of this study is to investigate the risk for cancer development in FMF.

**Methods:**

The risk ratio (RR) for malignancies was separately compared between FMF patients and fibromyalgia subjects, Still’s disease patients and Behçet’s disease patients. Clinical variables associated with cancer development in FMF patients were searched through binary logistic regression.

**Results:**

580 FMF patients and 102 fibromyalgia subjects, 1012 Behçet’s disease patients and 497 Still’s disease patients were enrolled. The RR for the occurrence of malignant neoplasms was 0.26 (95% Confidence Interval [CI.] 0.10-0.73, p=0.006) in patients with FMF compared to fibromyalgia subjects; the RR for the occurrence of malignant cancer was 0.51 (95% CI. 0.23-1.16, *p*=0.10) in FMF compared to Still’s disease and 0.60 (95% CI. 0.29-1.28, *p*=0.18) in FMF compared to Behçet’s disease. At logistic regression, the risk of occurrence of malignant neoplasms in FMF patients was associated with the age at disease onset (β1 = 0.039, 95% CI. 0.001-0.071, *p*=0.02), the age at the diagnosis (β1 = 0.048, 95% CI. 0.039-0.085, *p*=0.006), the age at the enrolment (β1 = 0.05, 95% CI. 0.007-0.068, *p*=0.01), the number of attacks per year (β1 = 0.011, 95% CI. 0.001- 0.019, *p*=0.008), the use of biotechnological agents (β1 = 1.77, 95% CI. 0.43-3.19, *p*=0.009), the use of anti-IL-1 agents (β1 = 2.089, 95% CI. 0.7-3.5, *p*=0.002).

**Conclusions:**

The risk for cancer is reduced in Caucasic FMF patients; however, when malignant neoplasms occur, this is more frequent in FMF cases suffering from a severe disease phenotype and presenting a colchicine-resistant disease.

## Introduction

Familial Mediterranean fever (FMF) represents the archetype of autoinflammatory diseases. It is caused by mutations in the *MEFV* gene, which encodes for the pyrin protein, an essential component of the NLRP3-inflammasome, an intracellular multiprotein complex responsible for the activation of the pro-inflammatory cytokines interleukin (IL)-1 and IL-18 (1, 2). It is clinically characterized by recurrent fever attacks typically lasting less than 72 hours and variably associated with serositis, severe abdominal pain (owing to peritonitis), arthritis, and erysipela-like erythema. However, many other tissues may be involved with inflammation leading to different phenotypes (3). Inflammatory attacks are associated with a pronounced increase in the laboratory inflammatory markers, especially serum amyloid A (4). Amyloidosis develops in not adequately treated patients over time (5).

Inflammation may be highly linked to cancer development, while IL-1 has been found to be correlate with the advanced and aggressive nature of some neoplasms (6). In this perspective, FMF should be burdened by an increased risk for malignancies. On the other side, NLRP3 inflammasome–mediated inflammatory cytokines may have a protective role in some cancers, including colorectal cancer, hepatocellular carcinoma and melanoma (7). Therefore, assuming an increased risk of cancer in FMF patients based solely on the inflammatory nature of this disease could be an incorrect hypothesis. Actually, some studies based on national enrolment have disclosed a reduced risk for cancer in FMF patients (8–10). The present study is thought to investigate this condition at a supranational level using data collected from the international AutoInflammatory Disease Alliance (AIDA) Network registry dedicated to monogenic autoinflammatory diseases (11). This approach aims to overcome influences related to the living environment and different social behavior. In addition, this study is also thought to assess the risk for cancer compared to diseased controls with other systemic inflammatory disorders.

## Material and methods

Data related to FMF patients, including demographics, clinical and laboratory aspects, therapeutic information and neoplasms history, were drawn from the international AIDA Network registry dedicated to monogenic autoinflammatory diseases (11).

Patients with Still’s disease and Behçet’s disease were included as controls affected by different systemic inflammatory disorders and their data were drawn from the international AIDA Network registries dedicated to Still’s disease and Behçet’s disease, respectively (12, 13). Consecutive fibromyalgia subjects not affected by other systemic inflammatory conditions were chosen as control group, taking care to include patients of different ethnicities, and matching for the sex with FMF patients. This choice was determined by the need to compare patients with FMF to individuals affected by a clinical condition completely devoid of inflammatory disorders.

The follow-up period ranged from the start of FMF, Still’s disease and Behçet’s disease to the last visit collected in the AIDA Network registries (up to January 2024). Consequently, this is an ambidirectional cohort study.

The primary aim of the study was to describe the frequency of malignant neoplasms in FMF patients and then to compare the risk versus fibromyalgia subjects, Still’s disease and Behçet’s disease. The secondary aim was to assess variables associated with the risk of neoplasms in the subgroup of FMF patients developing malignant cancer.

The endpoint of the study was the occurrence of malignant neoplasms during the whole follow-up period; the occurrence of neoplasms was reported in the AIDA network registries according to the international classification of diseases, version 10 (ICD-10).

Inclusion criteria for patients with FMF consisted of the identification of at least one pathogenic or likely pathogenic *MEFV* variant together with the fulfillment of the Eurofever classification criteria for FMF (14); alternatively, patients had to fulfill clinical diagnostic criteria for FMF proposed by Livneh et al. (15). Pathogenicity of *MEFV* variants was derived from Infevers, an online database for autoinflammatory mutations. Copyright. Available at https://infevers.umai-montpellier.fr/ (16–19).

Still’s disease patients fulfilled the classification criteria proposed by Yamaguchi et al. (20) and/or Fautrel et al. (21), while Behçet’s disease fulfilled the International Study Group criteria (22) and/or the International Criteria for Behçet’s disease (23). Classification criteria for pediatric Behçet’s disease were also applied to patients aged < 16 years (24). Fibromyalgia was classified according to either the American College of Rheumatology 1990 Criteria or American College of Rheumatology preliminary diagnostic criteria proposed by Wolfe et al. (25).

All patients included in this study gave their informed consent to participate. The study protocol was conformed to the tenets of the Declaration of Helsinki and was approved by the Ethics Committee of the Azienda Ospedaliero-Universitaria Senese, Siena, Italy in June 2019 (Ref. N. 14951).

Data analysis included the following descriptive statistics: sample sizes, percentages, frequency counts, mean, median, standard deviations and interquartile range calculations. The relative risk (RR) for cancer development between study groups and the corresponding 95% confidence intervals (95% CI.) were calculated by using the Episheet software considering the number of patients with cancer (cases) and the total number of observations among patients with FMF (exposed group) and controls (unexposed groups) (26). The Chi-square test or the Fisher exact test were used for pairwise comparisons of qualitative data; the Kruskall-Wallis test was used for multiple comparisons of quantitative data after having proved their non-gaussian distribution with the Shapiro-Wilk test. The t-test or the Mann-Whitney U test were used, as required, for investigating pairwise comparisons for quantitative data and for post-hoc analysis; in this last case, Bonferroni correction was also applied. Univariate binomial logistic regression was used to investigate any association between neoplasm development and the following variables: age at disease onset, age at diagnosis, age at enrollment, disease duration at diagnosis, tabagism, specific *MEFV* mutations, amyloidosis development, signs and symptoms during fever attacks (thoracic pain, pericarditis, pleuritis, abdominal pain, arthritis, spondylarthritis, skin manifestations, lymphadenopathy), colchicine treatment duration, use of biotechnological agents and use of anti-IL-1 agents. The β0 and β1 estimates were provided from logistic regressions with the exponential of β0 corresponding to the odds of cancer development when the variable is equal to zero. The corresponding RR were also calculated using the inverse-logit function of (β0 + β1) divided by the inverse-logit function of β0. Two-tailed statistical analyses were conducted, with a type I error set at 0.05 (*p* < 0.05), using RStudio software version 4.3.0.

## Results

In total, 580 FMF patients were enrolled; their demographic and clinical data are summarized in **Tables 1**, **2**. In addition, 102 fibromyalgia subjects, 1012 Behçet’s disease patients and 497 Still’s disease patients were enrolled as controls.

**Table 1:** Demographics of patients enrolled, distinguished according to the specific diseases they suffer from.

	FMF (n=580)	Fibromyalgia subjects (n=102)	Still’s disease (n=497)	BD (n=1012)	*p*-value
**Sex (n female/male)**	315/264	55/47	288/207	449/559	<0.001^c,f^
**Age at disease onset, years**	6.4 (IQR 12.3)	35.1 ± 16.1 (SD)	32.4 ± 17.3 (SD)	26.8 (IQR 17.1)	<0.0001^a,b,c,e,f^
**Age at diagnosis, years (mean ± SD)**	20.5 ± 16.4	43.07 ± 14.8	33.7 ± 17.4	33.6 ± 31.7	<0.0001^a,b,c,d,e^
**Disease duration at enrollment, years median (IQR)**	17.2 (22.7)	6 (14)	4.9 (7.7)	10.1 (13.1)	<0.0001^a,b,c,e,f^
**Age at enrolment, years (mean ± SD)**	30.9 ± 17.4	45.53 ± 15.4	39.7± 18.2	41.4 ± 33.4	<0.0001^a,b,c,d^
Ethnic origin					
• **Caucasic** **• Arab** **• Asian** **• Hispanic** **• Jew** **• Black** • **Others** **• Missing**	384 (66.2)	68 (66.7)	352 (70.8)	616 (60.9)	0.001^f^
	99 (17.1)	17 (16.7)	48 (9.7)	275 (27.2)	<0.0001^b,c,f^
	9 (1.6)	2 (2)	3 (0.6)	3 (0.3)	0.02
	5 (0.9)	1 (0.9)	42 (8.5)	16 (1.5)	<0.0001^b,f^
	3 (0.5)	0	1 (0.2)	1 (0.1)	0.37
	3 (0.5)	1 (0.9)	7 (1.4)	18 (1.8)	0.18
	13 (2.2)	2 (2)	4 (0.8)	0	<0.0001^c^
	64 (11)	11 (10.8)	40 (8)	83 (8.2)	0.19

Global p-values were obtained with Kruskal-Wallis test or ANOVA test for quantitative data and with chi-square test for qualitative data. Post hoc analysis was performed with the chi-square test or with the Fisher Exact test according to the expected frequencies; the letter “a” refers to a statistically significant differences between the FMF group and fibromyalgia group; the letter “b” refers to a statistically significant differences between the FMF group and Still’s disease group; the letter “c” refers to a statistically significant differences between the FMF group and the Behçet’s disease group; the letter “d” refers to a statistically significant difference between fibromyalgia subjects and Still’s disease; the letter “e” refers to a statistically significant difference between fibromyalgia subjects and Behçet’s disease; the letter “f” refers to a statistically significant difference between Still’s disease and Behçet’s disease. Post-hoc analysis was performed using the Bonferroni correction. Abbreviations: BD, Behçet disease; FMF, Familial Mediterranean Fever; IQR, interquartile range; n, number; SD, standard deviation.

**Table 2:** Frequency of clinical features associated to entire cohort of patients affected by Familial Mediterranean Fever (FMF) disease.

FMF disease manifestations (n=580)	
Disease course	
** •Relapsing-remitting, n (%)** ** • Chronic, n (%)** ** • Unknown, n (%)**	538 (92.7) 27 (4.7) 15 (2.6)
**Number attacks/year, median (IQR)**	12 (18)
**Mean duration of attacks (days), median (IQR)**	3 (1)
**Highest body temperature reached during attacks (°C), mean ± SD**	38.8 ± 32.8
**Thoracic pain, n (%)**	191 (32.9)
**Pericarditis, n (%)**	12 (0.02)
**Pleuritis, n (%)**	105 (18)
**Pleuropericarditis, n (%)**	7 (1.2)
**Abdominal pain with peritonitis, n (%)**	191 (32.9)
**Abdominal pain without peritonitis, n (%)**	138 (23.8)
**Vomiting, n (%)**	92 (15.9)
**Diarrhea, n (%)**	111 (19.1)
**Pharyngitis, n (%)**	61 (10.5)
**Oral aphthosis, n (%)**	72 (12.4)
**Genital aphthosis, n (%)**	5 (0.86)
**Erisipela-like skin rash, n (%)**	38 (6.6)
**Lymphadenopathy, n (%)**	31 (5.3)
**Splenomegaly, n (%)**	21 (3.6)
**Hepatomegaly, n (%)**	15 (2.6)
**Orchitis, n (% male patients)**	7 (2.9)
**Myalgia, n (%)**	148 (25.5)
**Arthralgia, n (%)**	234 (40.3)
**Arthritis, n (%)**	115 (19.8)
Type of arthritis	
** • Monoarthritis, n (%)** ** • Oligoarthritis, n (%)** ** • Polyarticular, n (%)**	28 (4.8) 75 (12.9) 7 (1.2)
**Spondyloarthritis, n (%)**	18 (3.1)
**Conjunctivitis, n (%)**	19 (3.3)
**Periorbital oedema, n (%)**	8 (1.4)
**Seizures, n (%)**	22 (3.8)
**Aseptic meningitis, n (%)**	2 (0.3)

Relapsing-remitting describes a disease course characterized by acute episodes interspersed with phases of well-being, whereas a chronic course pertains to patients who do not experience complete clinical or laboratory suppression of inflammation. Abbreviations: FMF, Familial Mediterranean Fever; IQR, interquartile range; n, number; SD, standard deviation.

In 14 (2.4%) FMF patients the following neoplasms were reported: monoclonal gammopathy (n=3), melanoma (n=3), thyroid carcinoma (n=3), basal cell carcinoma of skin (n=2), a benign neoplasm of transverse colon, a malignant neoplasm of testis, an adenocarcinoma of the cervix, one angiomyolipoma, one hepatic hemangioma, one uterine myoma, one prolactinoma. Eight neoplasms could be considered benign (monoclonal gammopathy, angiomyolipoma, hepatic hemangioma, uterine myoma, prolactinoma, in addition to the benign neoplasm of transverse colon). Three out of the 14 patients with tumors suffered from 5 different neoplasms; in particular, 2 patients with monoclonal gammopathy also suffered from other neoplasms (basal cell carcinoma and thyroid carcinoma); the patient with benign neoplasm of transverse colon also suffered from melanoma. Demographic, clinical and therapeutic details from the 9 patients with malignancies are reported in **Table 3**.

**Table 3:** demographic, clinical, genetic and treatment information from the nine familial mediterranean fever patients with malignancies.

Patients, n	1	2	3	4	5	6	7	8	9
**Neoplasm**	Adenocarcinoma of the cervix	Basal cell carcinoma of skin; MGUS	Thyroid carcinoma	Melanoma in situ; basal cell carcinoma of skin	Thyroid carcinoma; MGUS	Melanoma; benign neoplasm of transverse colon	Thyroid carcinoma	Malignant neoplasm of testis	Melanoma
**Age at enrolment, years**	49.1	44.8	58.2	49.3	64.7	61.1	39.3	34.8	2
**Age at onset, years**	36.1	33.2	43.8	39.2	12.5	15.9	5	4.2	0.5
**Disease duration, years**	10	11.6	14.4	10.1	52.2	45.2	34.3	30.6	1.5
**Sex**	Female	Male	Male	Female	Female	Male	Male	Male	Male
**Ethnicity**	Caucasic	Caucasic	Caucasic	Not provided	Caucasic	Caucasic	Caucasic	Caucasic	Caucasic
**Frequency of attacks/year**	Chronic course	14	12	12	12	12	4	12	Not provided
**Mutations**	Clinically determined diagnosis	R761H (Het) P268S (Het)	P369S (Het)	E148Q (Het) R761H (Het)	V726A (Hom)	M694V (Het)	Clinically determined diagnosis	M694V (Hom)	M694V (Het)
**Comorbidities**	Type 1 diabetes; allergic asthma; Hashimoto’s thyroiditis; gastroesophageal reflux disease; fibromyalgia; right incomplete bundle branch block	Chronic sinusitis; light tricuspid valve insufficiency; phimosis	Essential (primary) hypertension	None	Previous tuberculosis infection; chronic viral hepatitis B with delta-agent	Nontoxic single thyroid nodule	None	Essential (primary) hypertension	None
**Treatments performed**	Antihistamines; colchicine; anakinra	Colchicine	Colchicine, anakinra	Colchicine	Colchicine; anakinra; canakinumab	Colchicine	Colchicine; anakinra	Colchicine; anakinra; canakinumab	Colchicine

Het, heterozygous; Hom, homozygous; MGUS, monoclonal gammopathy of uncertain significance.

**Table 4** details the *MEFV* mutations observed in the patients with cancer in the current cohort of FMF; in 2 patients the diagnosis was clinically determined according to Livneh criteria (15).

**Table 4:** *MEFV* gene mutations observed in familial Mediterranean fever patients who have developed the two more frequent cancers and the other malignant neoplasms in the current FMF cohort.

Neoplasm	*MEFV* mutations (n patients)
**Melanoma**	• Heterozygous M694V (n=2) • Heterozygous R761H (n=1) • Heterozygous E148Q (n=1)
**Thyroid carcinoma**	• Heterozygous P369S (n=1) • Homozygous V726A (n=1) • No mutation searched (n=1)
**Other cancers**	• Heterozygous R761H (n=1) • Heterozygous P268S (n=1) • Homozygous M694V (n=1) • No mutation searched (n=1)

n, number.

Six (5.9%) neoplasms were observed among fibromyalgia subjects, 19 (3.8%) among Still’s disease patients (4 of which to be considered benign), 43 (4.2%) among Behçet’s disease patients (17 of which to be considered benign).

**Figure 1:**
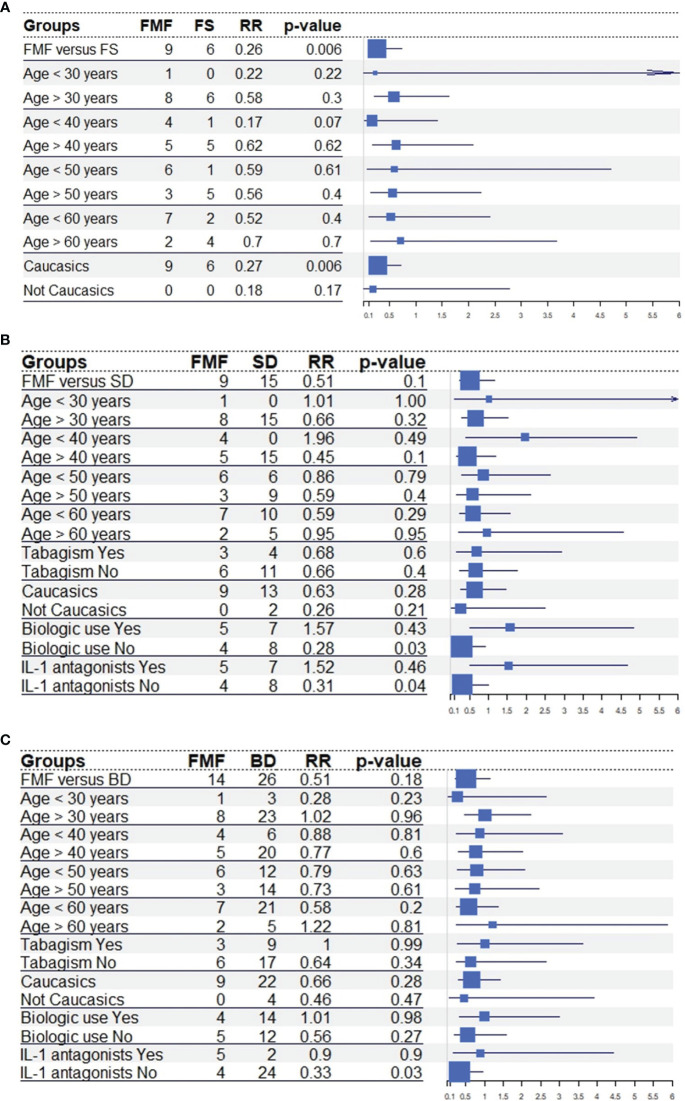
Forestplots illustrating the risk ratio (RR) for malignant cancers between familial Mediterranean fever patients and fibromyalgia subjects **(A)**, Behçet’s disease **(B)** and Still’s disease **(C)** in the total number of patients, in different age groups and according to the smoking habit and the use of biotechnological agents. Tabagism was not investigated toward fibromyalgia subjects due to the lack of this information in patients with fibromyalgia; biotechnological agents and anti-interleukin-1 agents were not investigated in patients with Behçet’s disease as patients with familial Mediterranean fever were primarily treated with anti-IL-1 agents, while Behçet’s disease was predominantly treated with tumor necrosis factor inhibitors. Risk Ratios and 95% confidence intervals were calculated with Episheet software; the p-value were obtained with the chi-square test or the Fisher exact test, as appropriate. Abbreviations: BD, Behçet’s disease; FMF, familial mediterranean fever; FS, fibromyalgia subjects; IL-1, interleukin-1; RR, risk ratio; SD, Still’s disease.

The RR to observe a malignant neoplasm was 0.26 (95% CI. 0.10-0.73, *p*=0.006) in FMF patients compared to fibromyalgia subjects, 0.51 (95% CI. 0.23-1.16, *p*=0.10) in FMF patients compared to Still’s disease and 0.60 (95% CI. 0.29-1.28, *p*=0.18) in FMF patients compared to Behçet’s disease. **Figure 1** shows the RR adjusted for the age of patients at enrolment, tabagism and treatment with biotechnological agents.

At univariate binary logistic regression, the occurrence of malignant neoplasia was associated with the age at disease onset (β1 = 0.039, 95% CI. 0.001-0.071, *p*=0.02), the age at the diagnosis (β1 = 0.048, 95% CI. 0.039-0.085, *p*=0.006), the age at the enrolment (β1 = 0.05, 95% CI. 0.007-0.068, *p*=0.01), the number of attacks per year (β1 = 0.011, 95% CI. 0.001- 0.019, *p*=0.008), the use of biotechnological agents (β1 = 1.77, 95% CI. 0.43-3.19, *p*=0.009), and the use of anti-IL-1 agents (β1 = 2.089, 95% CI. 0.7-3.5, *p*=0.002). In this last regard, patients requiring the use of biotechnological agents due to colchicine effectiveness issues were 4 out of 9 among FMF patients with malignant cancer development and 71 out of 571 among patients with no cancer occurrence (*p*=0.02). All FMF patients with malignant neoplasms and treated with biotechnological agents during their history had experienced cancer before starting biologic drugs.

**Table 5** presents β0 estimates from univariate binary logistic regression for variables significantly associated with the occurrence of malignant neoplasms, along with the corresponding RR values and their interpretation. **Table 6** displays β1 estimates and *p*-values for additional variables evaluated in this study, which, however, did not reach statistical significance. **Table 7** provides information about biotechnological treatment in enrolled patients.

**Table 5:** The exponential of β0 estimates obtained from the univariate binomial logistic regression for variables significantly associated with malignancy in familial Mediterranean fever (FMF) patients.

	exp(β0)=Odds	RR	*p*-value	Interpretations corresponding
**Age at disease onset**	Odds=0.007, 95% CI. 0.002-0.019	RR=1.04	0.02	The risk of developing cancer increases by 4% per year
**Age at the diagnosis**	Odds=0.003, 95% CI. 0.0005-0.012	RR=1.05	0.006	The risk of developing cancer increases by 5% per year
**Age at the enrollment**	Odds=0.003, 95% CI. 0.0004-0.016	RR=1.05	0.01	The risk of developing cancer increases by 5% per year
**Use of biotechnological agents**	Odds=0.008, 95% CI. 0.002-0.020	RR=5.7	0.009	A 470% higher risk of developing cancer has been found in patients requiring the use of biotechnological agents compared to those not requiring biologics
**Use of anti-IL-1 agents**	Odds=0.008, 95% CI. 0.002-0.019	RR=7.6	0.002	A 660% higher risk of developing cancer was observed in patients undergoing anti-IL-1 agents compared to patients not requiring this treatment
**N of attacks per year**	Odds=0.011, 95% CI. 0.005-0.023	RR=1.11	0.008	The increase of one FMF attack per year leads to an increase of 11% in the risk of malignant occurrence

The corresponding relative risk (RR) values and their interpretation have also been provided. RR values were calculated using the inverse-logit function of (β0 + β1) divided by the inverse-logit function of β0. Abbreviations: CI., confidence interval; EXP, exponential; IL-1, interleukin-1; N, number.

**Table 6:** The β1 estimates obtained from univariate binary logistic regression for variables assessed in this study, but without reaching statistical significance.

Variables	β1 estimate	*p*-value
**Disease duration at diagnosis**	0.035	0.14
**Tabagism**	0.625	0.398
**M694V mutation**	-0.069	0.92
**R761H mutation**	1.557	0.054
**E148Q mutation**	-0.001	0.99
**V726A mutation**	-0.03	0.93
**P268S mutation**	18.354	0.98
**P369S mutation**	1.412	0.19
**Amyloidosis development**	0.566	0.61
**Disease course (chronic versus relapsing-remitting)**	-0.935	0.39
**Thoracic pain**	1.19	0.10
**Skin manifestations**	1.360	0.10
**Lymphoadenopathy**	0.438	0.68
**Pericarditis**	1.367	0.21
**Pleuritis**	-0.585	0.58
**Abdominal pain**	-0.809	0.32
**Arthritis**	0.344	0.64
**Increased ESR**	17.09	0.99
**Increased CRP**	0.213	0.84
**Spondyloarthritis**	1.057	0.07
**Colchicine treatment duration**	-0.0004	0.905

ESR, Erythrocyte sedimentation rate; CRP, C reactive protein.

**Table 7:** Use of biotechnological agents in enrolled patients.

	FMF (n=580)	Still’s disease (n=497)	BD (n=1012)
**Biotechnological treatments**	104 (17.9%)	229 (46.1%)	369 (36.5%)
Anti-IL-1 agents	
** • Anakinra** ** • Canakinumab**	56 (9.6%) 38 (6.5%)	130 (26.1%) 70 (14.1%)	26 (2.6%) 8 (0.8%)

The variable “Biotechnological agents” encompasses both tumor necrosis factor inhibitors and interleukin blockers, including anti-IL-1 agents. Abbreviations: BD, Behçet’s disease; FMF, familial mediterranean fever; IL-1, interleukin-1.

## Discussion

The link between chronic inflammation and cancer is supported by strong evidence (27). On the other hand, innate immunity has been also described as a potential weapon against neoplasms, particularly through natural killer cells, macrophages, and eosinophils (28). In this scenario, FMF proves to be both a typical example of a systemic inflammatory disorder and the archetype of autoinflammatory diseases, precisely caused by dysregulation of the innate immune system (29, 30). For this reason, studying the oncological risk in FMF patients is particularly intriguing, as there is both a potential increase in cancer risk due to the chronic exposure to systemic inflammation and a possible decrease related to the hyperactivation of the innate immune system. In this regard, previous studies specifically assessing Turkish and Israelian populations have found a significant decrease in the cancer risk compared to general population (8–10). Accordingly, based on a multicentric data collection, the present study confirms a low frequency of neoplasms in FMF patients, which was equal to 1.5%. This frequency was even lower than that reported by Brenner et al. (8), corresponded to that reported by Baspinar et al. (9), and was slightly higher than that identified by Bilgin et al. (10).

Conversely, Twig et al. did not find a statistically significant difference in the hazard ratio for the occurrence of malignant disease of any type in FMF patients to control group; however, the analysis was performed in men subjects before the age of 50 years, among which the frequency of neoplasms was quite low in FMF patients (roughly 1%) (31).

The RR for cancer was reduced in FMF in all cases, both after comparison with fibromyalgia subjects and after comparison with controls affected by inflammatory diseases. However, statistical significance was achieved only towards fibromyalgia subjects as an example of non-inflammatory condition; in this case, the RR for malignant cancer was reduced of up to 74%. Nevertheless, the overall risk of encountering malignant neoplasms was reduced even when FMF was compared to controls suffering from inflammatory disorders, with a non-statistically significant risk reduction of 49% *versus* Still’s disease and 40% *versus* Behçet’s disease. Noteworthy, based on ethnic stratification in our data analysis, it appears that this phenomenon exhibits a stronger manifestation in Caucasian patients compared to individuals of other ethnic backgrounds. However, this discrepancy could be attributed to either real ethnic disparities or the limited representation of non-Caucasic patients in our study cohort. To address this ambiguity, we advocate for conducting a further study specifically focused on non-Caucasic patients in the near future. This would allow for a more comprehensive understanding of the phenomenon across diverse ethnic groups.

The presence of inflammation does not seem to increase the risk of malignant neoplasms in FMF, but the activity and severity of FMF might influence oncological risk. In this regard, logistic regression disclosed a significant association of malignant neoplasms in FMF patients with the age at disease onset and the number of inflammatory attacks per year. This seems to suggest that more severe phenotypes, frequently occurring in earlier stages of life with a higher frequency of attacks (32–35), may increase oncological risk among FMF patients. In this framework, the significant association between the occurrence of neoplasms and the use of biotechnological agents, including IL-1 inhibitors (**Figure 1**), observed towards Still’s disease and Behçet’s disease may lie. Indeed, neoplasms described in this study occurred before starting biotechnological agents in all FMF cases, while colchicine was associated to biotechnological agents due to effectiveness issues in roughly a half of patients with malignancy. This suggests that individuals necessitating biotechnological agents due to disease activity with treatment resistance are the same exhibiting a higher exposure to neoplasms. Indeed, although neoplasms could have occurred both before and after the potential use of biotechnological agents, neoplasms occurred only before the use of biologics in this cohort of FMF patients enrolled regardless of the use of specific therapies. This further corroborates data about the excellent safety profile with IL-1 inhibitors in FMF patients and provides more information about the increased rate of incident malignancy among patients treated with biotechnological agents as a controversial topic (36, 37).

The reduced incidence of neoplasms in patients with FMF is not currently easy to explain. A role has been suggested for colchicine (38, 39), which represents the first-line therapy in FMF. However, we cannot analyze this hypothesis, as all FMF patients included in the study have taken colchicine throughout their clinical history. Therefore, obtaining a comparison group is not feasible. Nevertheless, we have investigated an association between neoplasms and the duration of colchicine therapy, without identifying statistically significant associations. Of note, in the relationship between the pro-oncogenic risk posed by systemic inflammation and the potential anti-tumoral action of the innate immunity, the latter component could play a predominant role in FMF patients. However, excessive disease activity seems to play a contrary role, favoring a greater tendency towards oncogenesis among the group of FMF patients. In this regard, the recurrent nature of inflammatory attacks, particularly when accompanied by colchicine resistance, could result in a sustained inflammatory environment that in turn could contribute to carcinogenesis. In particular, specific laboratory studies should be conducted to understand whether sustained release of IL-1β can promote tumor progression.

In addition to the age at disease-onset, both the age at diagnosis and the age at the enrolment positively associated with malignant cancer development. Therefore, FMF patients, fibromyalgia subjects and controls with inflammatory diseases were stratified according to the different ages at which they were enrolled in the study. Considering the variations in RR by age groups, the age at the enrolment seems to work as an effect modifier rather than a confounding factor. Specifically, the progression of the age at enrolment generally led to an increased risk of neoplasia when comparing FMF to fibromyalgia subjects, as expected. Conversely, the reduction in the risk of tumors was less pronounced in younger age groups when comparing FMF and Still’s disease patients below the age of 60; the same effect was observed when comparing FMF and Behçet’s disease patients aged 40-60 years. On the other hand, tabagism showed to be a mediator of neoplasm development, especially when comparing FMF and Behçet’s disease patients.

Of note, mesothelioma has been described as associated with FMF in the past years (40–44); nevertheless, mesothelioma did not appear among neoplasms observed in our cohort of patients. Conversely, monoclonal gammopathy was observed in 3 out of the 14 patients with benign and malignant cancer. This aligned with what was previously studied regarding the identification of a high frequency of *MEFV* gene mutations in patients with hematological neoplasms (45–47).

Limitations of the study include the relatively limited number of FMF patients enrolled and the lack of an extensive adjustment for all cancer-related health habits. In additions, control groups may appear quite different in their demographic features. This last issue is mainly related to the well-known early age at FMF disease onset compared to the onset of other diseases chosen as control groups. This also affects the disease duration, the age at the diagnosis, and the age at the enrolment into the study. However, we ruled out any influence by the age of patients at the time of the enrolment by adjusting the results for different ages of patients. “Furthermore, the median disease duration at the time of enrollment in the study (more than 17 years) was significantly longer among FMF patients compared to the control groups. This shows a longer exposure to inflammation and the diseased status for FMF subjects. If a pro-tumorigenic role of FMF were to emerge in FMF patients, this would be highlighted precisely due to the prolonged exposure. In any case, this study provides further evidence regarding the risk of neoplasms in FMF patients, particularly among Caucasians, while also examining differences with fibromyalgia subjects and controls with other inflammatory diseases.

In conclusion, the present study confirms that the risk for neoplasms is reduced in Caucasic FMF patients; considering the subgroup of FMF subjects with cancer, the occurrence of tumors is more pronounced among patients showing a severe disease phenotype and those with a colchicine-resistant disease.

## Data availability statement

The original contributions presented in the study are included in the article/supplementary material. Further inquiries can be directed to the corresponding author.

## Ethics statement

The studies involving humans were approved by Azienda Ospedaliero Universitaria Senese. The studies were conducted in accordance with the local legislation and institutional requirements. Written informed consent for participation in this study was provided by the participants’ legal guardians/next of kin.

## Author contributions

AV: Writing – review & editing, Writing – original draft. VC: Writing – original draft, Writing – review & editing. AT: Writing – review & editing, Supervision. GR: Supervision, Writing – review & editing. EB: Supervision, Writing – review & editing. PPo: Writing – review & editing. EA: Writing – review & editing. JS: Writing – review & editing. GC: Writing – review & editing. ADP: Writing – review & editing. DR: Writing – review & editing. AO: Writing – review & editing. AŞ: Writing – review & editing. FL: Writing – review & editing. GL: Writing – review & editing. MC: Writing – review & editing. MM: Writing – review & editing. AI: Writing – review & editing. PPS: Writing – review & editing. EV: Writing – review & editing. DY: Writing – review & editing. HK: Writing – review & editing. RK: Writing – review & editing. AL: Writing – review & editing. MG: Writing – review & editing. MS: Writing – review & editing. SSe: Writing – review & editing. HE: Writing – review & editing. SO: Writing – review & editing. NJ: Writing – review & editing. MK: Writing – review & editing. ADC: Writing – review & editing. CG: Writing – review & editing. GM: Writing – review & editing. AA: Writing – review & editing. SP: Writing – review & editing. MR: Writing – review & editing. JS: Writing – review & editing. FD: Writing – review & editing. IM: Writing – review & editing. SSi: Writing – review & editing. MFG: Writing – review & editing. NÇ: Writing – review & editing. MT: Writing – review & editing. AK: Writing – review & editing. JH-R: Writing – review & editing. PPa: Writing – review & editing. DO-B: Writing – review & editing. PB: Writing – review & editing. AR: Writing – review & editing. SC: Writing – review & editing. PS: Writing – review & editing. HG: Writing – review & editing. SG: Writing – review & editing. EW-S: Writing – review & editing. İV: Writing – review & editing. RL: Writing – review & editing. KJ-R: Writing – review & editing. EM: Writing – review & editing. JT: Writing – review & editing. ACa: Writing – review & editing. ACo: Writing – review & editing. GE: Writing – review & editing. FL: Writing – review & editing. GB: Writing – review & editing. RT: Writing – review & editing. PR: Writing – review & editing. ED: Writing – review & editing. ST: Writing – review & editing. ABr: Writing – review & editing. BO: Writing – review & editing. AH-A: Writing – review & editing. ABa: Writing – review & editing. CF: Writing – review & editing. BF: Writing – review & editing. LC: Writing – review & editing, Project administration, Supervision.
